# SCREW VS. SUTURE FIXATION FOR TIBIAL EMINENCE FRACTURES: A META-ANALYSIS

**DOI:** 10.1590/1413-785220263404e300987

**Published:** 2026-07-24

**Authors:** Paulo Eduardo Dias Lavigne, Marcus Vinicius Ribeiro, Victor Mendes Ribeiro, Jessiel Gonçalves de Souza, Adriano Assis Nascimento, Eiffel Tsuyoshi Dobashi

**Affiliations:** 1 Universidade Federal de Sao Paulo (EPM/UNIFESP), Escola Paulista de Medicina, Sao Paulo, SP, Brazil.

**Keywords:** Tibial Spine Fractures, Anterior Cruciate Ligament Injuries, Orthopedic Surgery, Arthroscopy, Internal Fixators, Meta-Analysis as Topic, Knee Joint, Fraturas da Espinha Tibial, Lesões do Ligamento Cruzado Anterior, Cirurgia Ortopédica, Artroscopia, Fixadores Internos, Meta-Análise como Assunto, Articulação do Joelho

## Abstract

**Objectives:**

To compare functional and surgical outcomes between screw and suture fixation in pediatric tibial spine fractures.

**Materials and Methods:**

This systematic review followed PRISMA guidelines and was registered in PROSPERO (CRD420251022233). Cohort studies comparing arthroscopic screw versus suture fixation were included. The primary outcome assessed was postoperative knee function.

**Results:**

Four retrospective cohort studies were included, totaling 186 patients. No significant differences were found in knee function (SMD = −0.07; p = 0.702). Operative time was significantly shorter in the screw group (MD = −9.17 minutes; p < 0.001). However, this group showed a significantly higher risk of reoperation (RR = 1.78; p = 0.040) and implant removal (RR = 6.25; p = 0.040). No differences were observed regarding joint instability (RR = 0.90; p = 0.770) or return to sport (RR = 1.83; p = 0.190). Funnel plot analysis suggested potential publication bias.

**Conclusion:**

Both techniques yielded similar postoperative functional outcomes. Screw fixation was associated with shorter operative time but higher complication rates. **Level of evidence IIa; systematic review of cohort studies.**

## RESUMO

**Objetivos:**

Comparar os desfechos funcionais e cirúrgicos entre parafuso e sutura na fixação de fraturas de espinha tibial em pacientes pediátricos.

**Material e Métodos:**

Esta revisão sistemática seguiu o guideline PRISMA, registrada no PROSPERO (CRD420251022233). Foram incluídos estudos de coorte que compararam, por via artroscópica, a fixação com parafuso versus sutura. O desfecho primário analisado foi verificar a função do joelho no período pós-operatório.

**Resultados:**

Foram incluídos quatro estudos de coorte retrospectivos, totalizando 186 pacientes. Não foram observadas diferenças significativas na função do joelho (DMP = −0,07; p = 0,702). O tempo operatório foi significativamente menor no grupo tratado com parafuso (DM = −9,17 minutos; p < 0,001). No entanto, esse grupo apresentou risco significativamente maior de reoperação (RR = 1,78; p = 0,040) e de remoção do implante (RR = 6,25; p = 0,040). Não foram encontradas diferenças em relação à instabilidade articular (RR = 0,90; p = 0,770) ou ao retorno à prática esportiva (RR = 1,83; p = 0,190). A análise do gráfico de funil sugeriu possível viés de publicação.

**Conclusão:**

Ambas as técnicas apresentaram resultados funcionais pós-operatórios semelhantes. A fixação com parafuso foi associada a menor tempo cirúrgico, porém com maiores taxas de complicações. **Nível de evidência IIa; revisão sistemática de estudos de coorte.**

## INTRODUCTION

Tibial spine fractures are caused by the avulsion of the anterior cruciate ligament (ACL) at its insertion on the intercondylar eminence, occurring more frequently in children. Although uncommon, such traumatic disorders have clinical relevance and require careful evaluation, especially in cases with displacement.^1,2^ The Meyers and McKeever classification remains the most commonly used reference to guide surgical indication in type II fractures with displacement, as well as in type III and IV fractures.^3^

The most commonly employed fixation techniques include the use of metal screws (SW) and non-absorbable suture fixation (SF), both frequently performed arthroscopically.^4^ These approaches aim to restore the anatomical alignment of the tibial spine, preserve ACL function, and enable a safe return to sports activities.^5^ The choice of technique depends on the fracture morphology, the patient's age, the desired degree of stability, and the surgeon's experience. However, uncertainty persists regarding the ideal surgical strategy.^6^

Biomechanical studies have reported conflicting results regarding mechanical strength between different fixation methods, and direct clinical evidence remains limited.^7,8^ Although both techniques have demonstrated satisfactory outcomes in clinical practice, it is still unclear whether one presents superiority in essential aspects such as knee function, complication rates, operative time, or return to sport.^9,10^

In the absence of randomized clinical trials (RCTs) and given the predominance of observational studies, this systematic review with meta-analysis and meta-regression aims to synthesize comparative observational data on screw versus suture fixation in tibial spine fractures. By analyzing functional and surgical outcomes in different clinical contexts, this study seeks to enhance the understanding of the patterns of results observed in practice, without the intention of establishing definitive recommendations, but rather to support a more informed surgical decision-making process.

## MATERIALS AND METHODS

This systematic literature review was conducted according to the guidelines of the Preferred Reporting Items for Systematic Reviews and Meta-Analyses (PRISMA)^11^. The primary objective was to compare the clinical and functional outcomes of screw fixation versus arthroscopic suture fixation in children and adolescents with tibial spine fractures.

The PICOTT strategy was applied as follows: Population (P) – Children and adolescents with tibial spine fractures/tibial eminence fractures/anterior cruciate ligament (ACL) avulsion fractures treated surgically; Intervention (I) – Screw fixation; Comparison (C) – Arthroscopic suture fixation; Outcomes (O) – Primary: Knee function and range of motion (ROM). Secondary outcomes: Return to sport, knee stability, postoperative complications, reoperation rates, time to radiographic bone consolidation; Study type (T) – Randomized clinical trials (RCTs) and cohort studies (prospective or retrospective); Time (T) – Any follow-up time.

This review was registered in the International Prospective Register of Systematic Reviews (PROSPERO) under registration number CRD420251022233. The methodology was defined and registered prior to the start of the search in the databases.

### Eligibility criteria

Eligible studies included full-text publications of RCTs, written in English and available until April 2025 in the PubMed, Scopus, Embase, and Cochrane CENTRAL databases. The studies should compare screw fixation and arthroscopic suture fixation, reporting clinical outcomes related to functional recovery and complications.

Articles not available in full text through digital platforms, conference abstracts, preprints (*preprints)*, letters to the editor, case reports and series, cross-sectional studies, and case-control studies, reviews, and studies without sufficient data on relevant outcomes were excluded. Duplicate publications of the same study were excluded, retaining only the most complete version.

### Search strategy

To search for articles in the databases, descriptors related to "Tibial Spine Fractures," "Screw Fixation," and "Arthroscopic Suture" were used. The descriptors were obtained from *Medical Subject Headings* (MeSH), accessed at www.ncbi.nlm.nih.gov/mesh/. Boolean operators AND and OR were employed to combine the terms on the mentioned platforms, respecting the inclusion and exclusion criteria of the articles. All descriptors used, as well as the complete search strategy for each database, are available in Supplementary Material 1 and 2. ^12,13^

### Study selection

Two independent reviewers, blinded to each other's assessments, jointly screened the titles and abstracts of all retrieved articles to identify those that met the previously established inclusion criteria. Potentially eligible studies were then read in full to confirm inclusion. In case of disagreement, the final decision was made by a senior reviewer, who had access only to the articles related to the research. The selection of studies was conducted using the Rayyan application.^14^ To ensure the comprehensiveness of the available literature, the snowballing strategy was also used:^15^ the reference lists of all relevant systematic reviews identified in the initial search were examined to identify additional eligible primary studies, and the references of the full-text articles were also analyzed after full reading to detect studies possibly not captured in the original search.

### Data extraction

Data extraction was performed independently and in duplicate (PEDL and JGS) to ensure the accuracy and reliability of the information. The data from the included studies were extracted using a pre-prepared form in Microsoft Excel® (version 2205), containing comprehensive information about the characteristics of the studies, including sample size, details of the intervention and control groups, methodological aspects, and the outcomes assessed. Any discrepancies between the reviewers were resolved by consensus or with the mediation of a senior reviewer (ETD).

### Quality Assessment

The assessment of bias risk was conducted based on the *Risk Of Bias In Non-randomized Studies - of Interventions* (ROBINS-I) tool proposed by Cochrane,^16^ applied independently by two reviewers (PEDL and JGS), with discrepancies resolved by consensus. Publication bias was assessed using a funnel plot with contour of statistical significance (*contour-enhanced funnel plot*), which displays point estimates based on the weight of each study and highlights areas of statistical significance to facilitate interpretation.^17^

### Statistical Analysis

The primary outcome of this meta-analysis was to verify knee function in the postoperative period. Given the use of distinct validated instruments among the studies (IKDC Subjective Scale and Lysholm score), this outcome was evaluated using the standardized mean difference (SMD), with 95% confidence intervals (CI95%). For operative time, uniformly measured in minutes, the mean difference (MD) was used. Dichotomous outcomes (such as knee instability in the postoperative period, failure to return to sport, reoperations, and implant removal) were analyzed using relative risk (RR), also with CI95%.

Heterogeneity was assessed with the Cochran Q test and the I^2^ index, with p values < 0.10 and I^2^ > 25% considered indicative of substantial heterogeneity. Prediction intervals (PI) were also reported to express the expected variation in effect sizes in similar future studies. Given the clinical and methodological variability among the included studies, all meta-analyses were conducted using a random effects model, applying the Restricted Maximum Likelihood (REML) estimator.

For the specific outcome of implant removal, due to the low frequency of these events and the limited number of studies, methodological adjustments were made to increase the robustness of the estimates: (1) continuity correction, by adding 0.5 to all cells of the contingency tables to account for data with zero events; and (2) confidence intervals were calculated using the Hartung-Knapp method,^18^ which is more conservative and recommended for meta-analyses with few studies and low event rates.

A sensitivity analysis leave-one-out was conducted for all outcomes to assess the influence of each individual study on the pooled estimates. For the primary outcome (function), an additional sensitivity analysis was conducted using the alternative Lysholm score reported by one of the studies. The funnel plot analysis was used to investigate potential publication bias in the primary outcome. Finally, meta-regression analyses were performed for the functional outcome to assess the influence of covariates such as the average age of participants, fracture severity (Meyers and McKeever classification), surgical access route (arthroscopic vs open), and sex distribution.

## RESULTS

**Figure 1:**
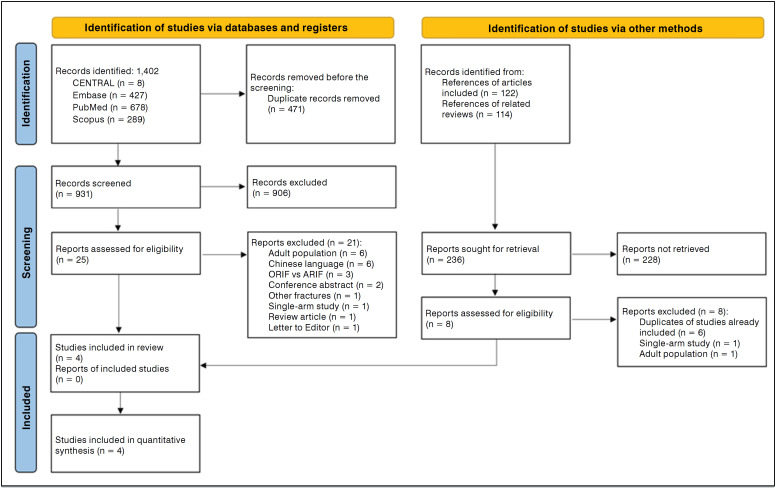
Flowchart of the included studies.

After the selection process, four articles were included in our study.^9,10,19,20^ The flowchart detailing the selection process of the studies and the reasons for exclusion can be found in Figure 1.

### Characteristics of the studies

Four retrospective cohort studies were included, conducted in South Korea^10^, the United States^9^, Europe (Finland, France, and Italy)^19^, and Turkey,^20^ with follow-up periods ranging from 1 to 2 years. In total, 186 patients were evaluated: 93 treated with screw fixation (SW) and 93 with suture fixation (SF). In the SW group, 63 patients were male (67.7%) and 30 were female (32.3%); in the SF group, 61 were male (65.6%) and 32 were female (34.4%). The average age was 14.5 ± 3.8 years in the SW group and 14.1 ± 4.1 years in the SF group.

All fractures were classified according to the Meyers and McKeever system.^9,10,19,20^ In the SW group, 1.1% were type I, 38.2% type II, and 60.1% type III. In the SF group, 29.0% were type II and 70.1% type III, with no cases of type I. The time between trauma and surgical intervention was reported in two studies (Seon et al., 2009^10^ and Ercan et al., 2024^20^), with an average of 13.13 ± 2.99 days in the SW group and 20.52 ± 3.51 days in the SF group. The arthroscopic technique was predominant in both groups, being used in 97.2% of SW cases and 96% of SF cases.

Three studies reported the postoperative immobilization protocol, with full extension adopted for all patients.^10,19,20^ The average duration of immobilization was 3.6 weeks, ranging from 2 to 4.5 weeks among the studies. More details of the included studies are presented in Table 1.

**Table 1:** Main characteristics of the included studies.

Study	Intervention		Design (Country)	Follow-up	Number of patients (M/F)		Age (years)		M&M Classification (I/ II/ III)		TIS (d)^†^		Surgical reduction approach (A/O)^‡^		Immobilization
	SW	SF			SW	SF	SW	SF	SW	SF	SW	SF	SW	SF	
Seon et al. (2009)	3.5–4.5 mm cannulated screw (1–2 units)	Non-absorbable suture	Retrospective cohort (South Korea)	2 years	10/6	13/04	17.7 ± 5.3	17.6 ± 7.2	0/6/10	0/5/12	29.8 ± 3.39	29.66 ± 3.34	32/ 3	24/4	Full extension with a long leg cast for 3 weeks
Callanan et al. (2019)	3.5–4.5 mm cannulated screw	Transosseous suture (Orthocord/PDS/FiberWire No. 2/5)	Retrospective cohort (EUA)	1 year	27/08	22/11	11.2 ± 3.2	12.4 ± 2.5	0/9/22	0/5/28	ND	ND	31/0	33/0	ND
Jääskelä et al. (2023)	SW (screw type, diameter or implant not specified)	Suture technique/type not specified	Retrospective cohort (Finland, France, and Italy)	2 years	17/12	18/14	11.5 ± 2.3	10.9 ± 2.7	1/26/34	0/12/20	ND	ND	29/0	32/0	In full extension for 4.5 weeks
Ercan et al. (2024)	Headless compression screw (diameter/material not specified)	Transosseous fixation with 2 Ultrabraid sutures	Retrospective cohort (Turkey)	2 years	9/4	8/3	10.8 ± 2.0	10.4 ± 2.1	0/5/8	0/5/6	6.0 ± 2.39	6.39 ± 3.76	13/0	12/0	In full extension with brace for 2 weeks.

Legends: SW = Screw fixation; SF = Suture fixation; M/F = Male/Female; M&M = Meyers and McKeever; I/II/III = Fracture classification I, II, III; TIS = Time from injury to surgery; d = days; A/O = Arthroscopic/Open; ND = Not described/Not defined; PDS = Polydioxanone; No. = Number. Mean ± Standard Deviation. Number of patients undergoing arthroscopy or open surgery.

### Meta-analysis of the included studies

#### Knee function

**Figure 2:**
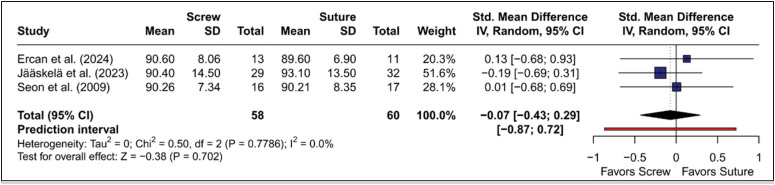
Forest plot of the meta-analysis for the functional outcome measured by the IKDC score.

Three studies were included for this outcome, totaling 118 patients. Jääskelä et al. (2023)^19^ and Ercan et al. (2024)^20^ used the subjective IKDC scale, while Seon et al. (2009)^10^ employed the Lysholm score to assess postoperative knee function. There was no statistically significant difference between the SW and SF groups (SMD = −0.07; 95% CI: −0.43 to 0.29; I2: −0.87 to 0.72; p = 0.702; I^2^ = 0%) (Figure 2).

The leave-one-out sensitivity analysis demonstrated that the exclusion of any individual study did not substantially alter the overall estimate, which remained statistically non-significant (Figure S1). A sensitivity analysis was also conducted using the Lysholm score additionally reported by Ercan et al. (2024),^20^ with equally consistent and non-significant results (SMD = 0.03; CI95%: −0.36 to 0.42; IQR: −0.99 to 1.04; p = 0.885; I^2^ = 17.8%) (Figure S2). The funnel plot analysis revealed an asymmetric distribution of studies, suggesting possible publication bias (Figure S3).

The meta-regression did not identify statistically significant associations between functional outcomes and the evaluated covariates: (1) mean age of participants (Figure S4 and Table S1); (2) mean severity of fracture according to Meyers and McKeever (Figure S5 and Table S2); (3) proportion of patients treated via arthroscopic or open methods (Figure S6 and Table S3); (4) proportion of male patients (Figure S7 and Table S4).

#### Operative time

Three studies, totaling 124 patients,^9,10,20^ were included for this outcome. The analysis, based on the average operative time in minutes, revealed a statistically significant difference in favor of the screw group (MD = −9.17; CI95%: –11.29 to −7.05; IQR: –13.82 to –4.52; p < 0.001; I^2^ = 66.1%) (Figure S8).

The leave-one-out sensitivity analysis showed that the exclusion of Seon et al. (2009)^10^ resulted in an MD of –8.93 minutes (CI95%: –11.42 to –6.44; I^2^ = 72.2%), while the exclusion of Ercan et al. (2024)^18^ generated an MD of –8.56 (CI95%: –12.70 to –4.42; I^2^ = 82.7%). On the other hand, the exclusion of Callanan et al. (2019)^9^ increased the effect size (MD = –29.45; CI95%: –45.97 to –12.93; I^2^ = 82.7%), while maintaining statistical significance in all scenarios (Figure S9).

### Postoperative complications

#### Knee instability

Three studies, totaling 125 patients, assessed postoperative knee instability through the Lachman test. Em Seon et al. (2009)^10^, patients with grade 1 or 2 were considered unstable, with no cases of higher grades. Ercan et al. (2024)^18^ included only patients with grade 1 instability. Callanan et al. (2019)^9^ described the cases as "unstable," without detailing the grades of the Lachman test. There was no statistically significant difference between the SW and SF groups (RR = 0.90; CI95%: 0.46 to 1.77; I^2^: 0.21 to 3.95; p = 0.770; I^2^ = 0%) (Figure S10). The sensitivity analysis confirmed the stability of the estimate (Figure S11).

#### Failure to return to sport

Three studies, with 162 patients,^9,10,19^ were included for this outcome. The analysis, based on the proportion of patients who did not return to sport, found no statistically significant difference between the groups (RR = 1.83; CI95%: 0.75 to 4.48; I^2^: 0.26 to 13.06; p = 0.190; I^2^ = 0%) (Figure S12). The sensitivity analysis *leave-one-out* confirmed the robustness of the findings (Figure S13).

#### Reoperation

Four studies, totaling 186 patients, were included.^9,10,19,20^ The analysis was based on the proportion of patients who required reoperation due to postoperative complications compared to those who did not require it. A statistically significant difference was observed between the groups, with a higher risk of reoperation in the SW group (RR = 1.78; 95% CI: 1.04 to 3.06; I^2^: 0.86 to 3.68; p = 0.040; I^2^ = 0%) (Figure S14). The sensitivity analysis *leave-one-out* confirmed the robustness of the findings, as the exclusion of any individual study did not substantially alter the overall estimate (Figure S15).

#### Implant removal

Three studies, with 125 patients, were included for review.^9,10,20^ The analysis was based on the proportion of patients who underwent implant removal compared to those who did not in each group. A statistically significant difference was observed, with a higher risk associated with the SW group (RR = 6.25; 95% CI: 1.19 to 32.90; IP: 0.70 to 55.79; p = 0.040; I^2^ = 0%) (Figure S16). The sensitivity analysis leave-one-out showed that the exclusion of Seon et al. (2009)^10^ or Callanan et al. (2019)^9^ did not substantially alter the overall estimate, which remained statistically significant. In contrast, the exclusion of Ercan et al. (2024)^18^ resulted in an even greater risk of implant removal in the SW group (RR = 7.21; 95% CI: 1.80 to 28.87; I^2^ = 0%) (Figure S17).

#### Assessment of the risk of bias of the included studies

**Figure 3:**
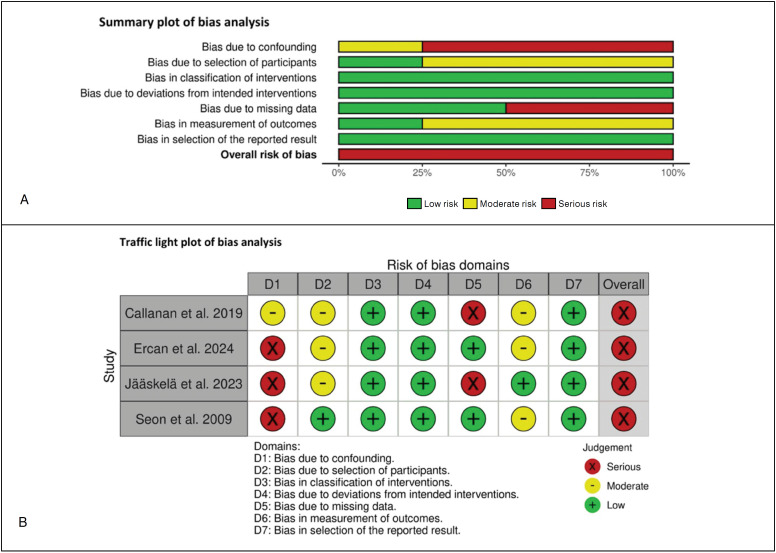
A and B. Summary and traffic graph of the bias analysis using the Risk of Bias in Non-Randomized Studies – of Interventions (ROBINS-I) tool.

The assessment of the risk of bias of the included studies, conducted with the Risk of Bias in Non-Randomized Studies of Interventions (ROBINS-I) tool, is summarized in Figures 3A and 3B.

#### Confounding bias

One study presented a moderate risk due to incomplete control of confounding factors^9^. Three studies were classified as having a severe risk: in Ercan et al. (2024)^20^, the type of fixation was determined by the year of surgery, introducing temporal bias and confusion related to the evolution of the technique; in Jääskelä et al. (2023)^19^, treatment varied by center and period, which may reflect institutional preferences and changes in practices; and in Seon et al. (2009)^10^, the choice of fixation method changed over time, raising concerns about confusion arising from changes in clinical practice.

#### Selection of participants

Seon et al. (2009)^10^ was considered to be of low risk. The other studies were classified as having a moderate risk. Callanan et al. (2019)^9^ e Ercan et al. (2024)^20^ excluded patients due to inadequate follow-up or associated injuries, which may have introduced selection bias. Jääskelä et al. (2023)^19^ presented a low response rate (53%), which may affect representativeness.

#### Interventions

All studies were assessed as having a low risk of bias regarding the classification of interventions and deviations from intended interventions (items 3 and 4 of ROBINS-I)^9,10,19.20^.

#### Missing data

Ercan et al. (2024)^20^ e Seon et al. (2009)^10^ were classified as low risk. Callanan et al. (2019)^9^ e Jääskelä et al. (2023)^19^ presented a serious risk due to high loss to follow-up, which may have influenced the results if the missing data were not random.

#### Outcomes

The article by Jääskelä et al. (2023)^19^ was considered low risk. The other three studies presented a moderate risk due to the lack of blinding and the assessment of subjective outcomes by the surgeon responsible for the treatment.^9,10,20^

#### Selective reporting

All studies were assessed as having a low risk for selective reporting of outcomes, with consistent presentation of the pre-planned outcomes.^9,10,19,20^

## DISCUSSION

This systematic review with meta-analysis demonstrated that, in pediatric patients with tibial spine fractures, both screw fixation and suture fixation result in comparable postoperative functional outcomes. However, screw fixation was associated with a shorter operative time, at the cost of a significantly higher risk of reoperation and implant removal. These findings proved robust in sensitivity analyses and were not significantly influenced by clinical variables such as age, sex, fracture severity, or surgical technique, as evidenced by meta-regression models. The funnel plot analysis for the primary functional outcome revealed an asymmetric distribution, with a notable absence of small studies favoring screw fixation. This pattern may suggest publication bias, particularly in contexts where neutral or favorable results for suture fixation are more frequently published. However, given the limited number of included studies, this evidence should be interpreted with caution as asymmetries in funnel plots with small samples may occur by chance. As highlighted in the Cochrane Handbook for Systematic Reviews of Interventions, funnel plots with fewer than 10 studies have limited reliability in detecting true publication bias^21^. Nevertheless, the wide prediction intervals observed suggest that the estimated effects may vary substantially in future studies, indicating that the differences between techniques may be modified as new evidence becomes available.

A previous systematic review supports the findings found in the present study. The review conducted by Chang et al. (2022)^22^ included five observational studies with 184 patients—primarily adolescents and young adults—and identified a higher incidence of reoperations and implant removals in the screw fixation group, despite no significant differences in functional outcomes. Additionally, high rates of return to high-impact sports were reported with suture fixation, even in type II to IV fractures, suggesting satisfactory long-term functional recovery. Although derived from an older population, these findings present points of convergence with the present analysis. The higher rates of reoperation and adverse events reported by Chang et al. (2022)^22^ may reflect anatomical, biomechanical, or scar response differences between older patients and children, reinforcing the need for specific studies for the pediatric population.

Gans et al. (2014)^23^, in a systematic review focused exclusively on pediatric patients, analyzed 451 cases of tibial eminence fractures. The authors reported that type III and IV fractures were associated with greater loss of range of motion, as well as a higher complication rate with screw fixation. On the other hand, suture fixation showed high rates of return to sports but was associated with adverse events in up to 30% of cases. In our research, no association was observed between the type of fracture and functional outcomes in the meta-regressions. Still, the findings of Gans et al. (2014)^23^ directly corroborate the results we found, regarding the functional equivalence between techniques and lower incidence of reoperations and complications with suture fixation. This consistency among studies strengthens the evidence that suture fixation presents a more favorable safety profile in this population, possibly due to the greater regenerative capacity and lower joint stiffness observed in children.

A randomized clinical trial with 90 young adults demonstrated superior functional performance with suture fixation, reflected in higher scores on the IKDC and Lysholm, along with the absence of reoperations in this group, in contrast to seven reinterventions in the screw group^24^. Similarly, Qu et al. (2022)^25^ reported a lower rate of adverse events with suture fixation in a cohort of 69 patients, although functional outcomes were similar between the groups. These findings reinforce the safety profile of suture fixation and suggest that such benefits may extend to age groups beyond the pediatric.

Single-arm observational studies also provide relevant insights. Yuan et al. (2015)^26^ and Çağlar et al. (2020)^27^, both with pediatric cohorts treated with suture fixation, reported good functional outcomes, low complication rates, and satisfactory return to activities. Also, Lutz et al. (2021)^28^, in a cohort of 23 patients, observed satisfactory ligament stability and return to high-impact sports, despite complications in 30% of cases. Regarding screw fixation, Shin et al. (2018)^29^ reported implant-related complications in 27 pediatric patients, while Wiegand et al. (2014)^30^ described preservation of short-term function in eight children treated with Herbert screws. Although these studies do not have comparative groups, their findings contribute to outlining the clinical profile and complications associated with each technique.

### Limitations of the study

This study has limitations that must be considered. All included articles are of the retrospective cohort type, with inherent risk of selection bias, confounding, and lack of standardization in data collection. We used the ROBINS-I tool to assess the risk of bias and conducted meta-regression analyses to explore potential sources of heterogeneity; however, the existence of residual confounding cannot be completely ruled out.

The small number of studies and participants included may have limited statistical power, especially for secondary outcomes. We employed conservative statistical approaches, including continuity correction, the Hartung-Knapp method, and prediction intervals to mitigate this impact. The study by Seon et al. (2009)^10^ included both adults and children, although the population was predominantly pediatric. The sensitivity analysis excluding this study did not show a significant change in the overall effect estimate. Furthermore, the meta-regression using the mean age of participants showed that age did not influence functional outcomes.

The absence of high-level randomized clinical trials limits the ability to establish causal relationships. However, given the scarcity of high-quality comparative data in this specific population, our synthesis of observational evidence provides relevant insights to support clinical practice. Finally, asymmetry was observed in the funnel plot for the functional outcome, suggesting possible publication bias. Due to the limited number of studies, the Egger test could not be applied; however, we employed funnel plots with significance contours to assist in interpretation.

Therefore, we adopted rigorous methodological practices, including prior registration of the protocol, independent and duplicate data extraction and evaluation, as well as multiple sensitivity analyses, which strengthens the reliability of our findings.

## CONCLUSION

This systematic review with meta-analysis demonstrated that suture fixation was associated with lower rates of reoperation and complications compared to screw fixation, while maintaining similar functional outcomes in the treatment of tibial spine fractures. These findings were consistent in sensitivity analyses and were not influenced by the clinical variables assessed through meta-regression. However, the prediction intervals suggest that these effects may vary with the publication of new studies, highlighting the presence of residual uncertainty. Although the available data indicate a potentially more favorable safety profile for suture fixation in pediatric patients, this evidence was based on observational studies. Better randomized clinical trials are needed to confirm these findings and guide the choice of the most appropriate surgical technique for tibial spine fractures in children.

## Data Availability

The materials underlying this research are publicly available on the Figshare platform, through the following DOIs: https://doi.org/10.6084/m9.figshare.31915674 referring to Supplemental Material 2, containing the complementary figures and tables; and https://doi.org/10.6084/m9.figshare.31915419, referring to Supplemental Material 1, containing the complete search strategy used in the review.
