# Supplementary material for: SCREW VS. SUTURE FIXATION FOR TIBIAL EMINENCE FRACTURES: A META-ANALYSIS
Source: Acta Ortop Bras. 2026 Jul 24;34(4):e300987. doi: 10.1590/1413-785220263404e300987 (PMC13399201; doi:10.1590/1413-785220263404e300987)
Supplement: Supplementary file 1 [file 1809-4406-aob-34-4-e300987-Suppl01.docx]

**SUPPLEMENTARY MATERIAL 1 – SEARCH STRATEGY**

**Title:** Screw versus Suture in the Fixation of Tibial Spine Fractures: A Systematic Review, Meta-Analysis, and Meta-Regression.

**Objective of Review**

The objective of this review is to compare the clinical and functional outcomes of screw fixation versus arthroscopic suture fixation in children and adolescents with tibial spine fractures

**PROSPERO ID:** CRD420251022233.

**Last review date in databases:** April 5, 2025.

**PICOTT Strategy**

Population – Children and adolescents with tibial spine fractures/tibial eminence fractures/anterior cruciate ligament (ACL) avulsion fractures treated surgically.

Intervention – Screw fixation

Comparison – Arthroscopic suture fixation

Outcomes – Primary: Knee function and Range of motion (ROM). Secondary: Return to sport, knee stability, postoperative complications, reoperation rates, time to radiographic bone healing.

Type of study – Randomized controlled trials and cohort studies (prospective or retrospective)

Time – Any length of follow-up, provided that clinically or functionally relevant outcomes are reported

**Keywords Used in Databases**

The study was conducted by integrating descriptors for Tibial Spine Fractures, Screw fixation, and Arthroscopic sutures. Descriptors from PubMed, Cochrane CENTRAL, Embase, and Scopus were sourced from the Medical Subject Headings (MeSH) (https://www.ncbi.nlm.nih.gov/mesh/) and supplemented with additional related terms.

**Database search strategy**

((Tibial Spine Fractures) OR (Tibial Eminence Fractures) OR (Intercondylar Eminence Fractures) OR (Tibial Spine Avulsion) OR (ACL avulsion fractures) OR (Anterior Cruciate Ligament Avulsion)) AND ((Screw) OR (Bone Screw) OR (Internal fixation)) AND ((Arthroscopic) OR (Arthroscopic suture) OR (Suture) OR (Sutur*))

**Search in each database**

**PubMed**

Total number of articles: 678

**Embase**

Total number of articles: 427

**Scopus**

Total number of articles: 289

**Cochrane CENTRAL**

Total number of articles: 8
