# Supplementary material for: SCREW VS. SUTURE FIXATION FOR TIBIAL EMINENCE FRACTURES: A META-ANALYSIS
Source: Acta Ortop Bras. 2026 Jul 24;34(4):e300987. doi: 10.1590/1413-785220263404e300987 (PMC13399201; doi:10.1590/1413-785220263404e300987)
Supplement: Supplementary file 2 [file 1809-4406-aob-34-4-e300987-Suppl02.docx]

**SUPPLEMENTARY MATERIAL 2 – FIGURES AND TABLES**

**Title:** Screw versus Suture in the Fixation of Tibial Spine Fractures: A Systematic Review, Meta-Analysis, and Meta-Regression.

**PROSPERO ID:** CRD420251022233.

**Figure S1.** Leave-one-out sensitivity analysis for the functional outcome (IKDC).


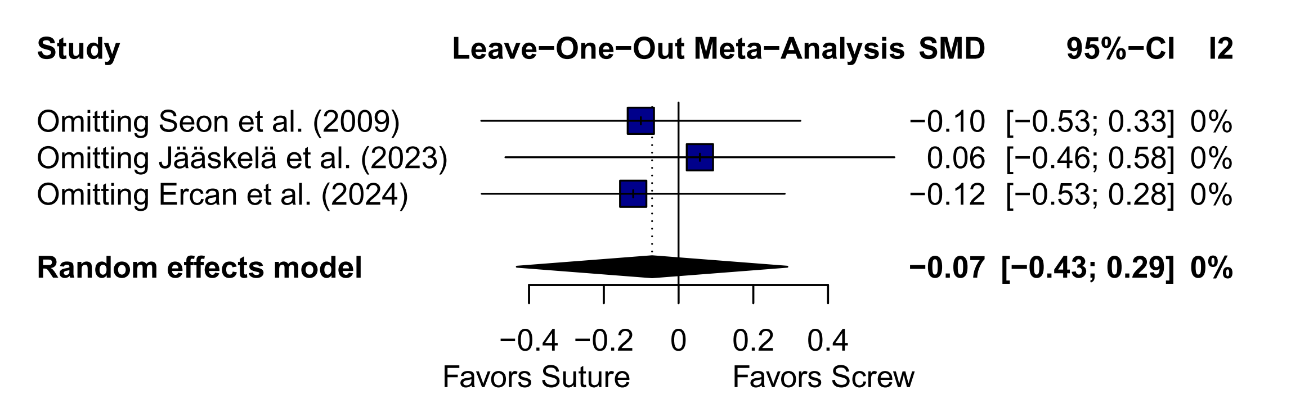


**Figure S2.** Sensitivity analysis of the Lysholm score as reported by Ercan et al. (2024).


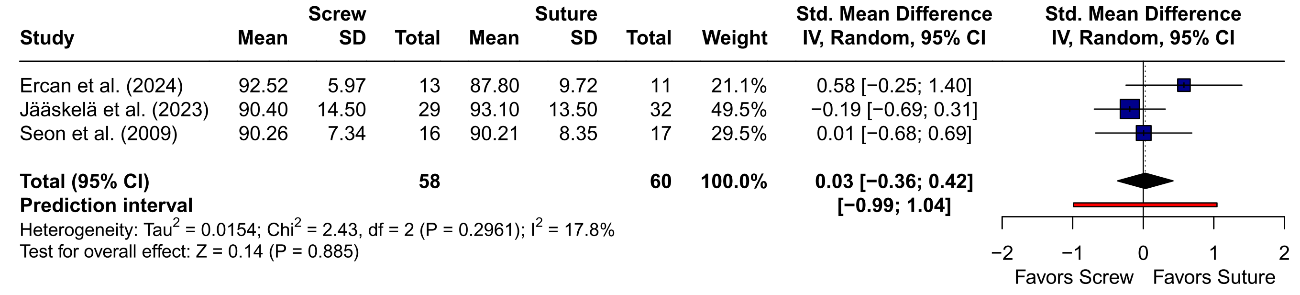


**Figure S3.** Contour-enhanced funnel plot for the primary functional outcome.


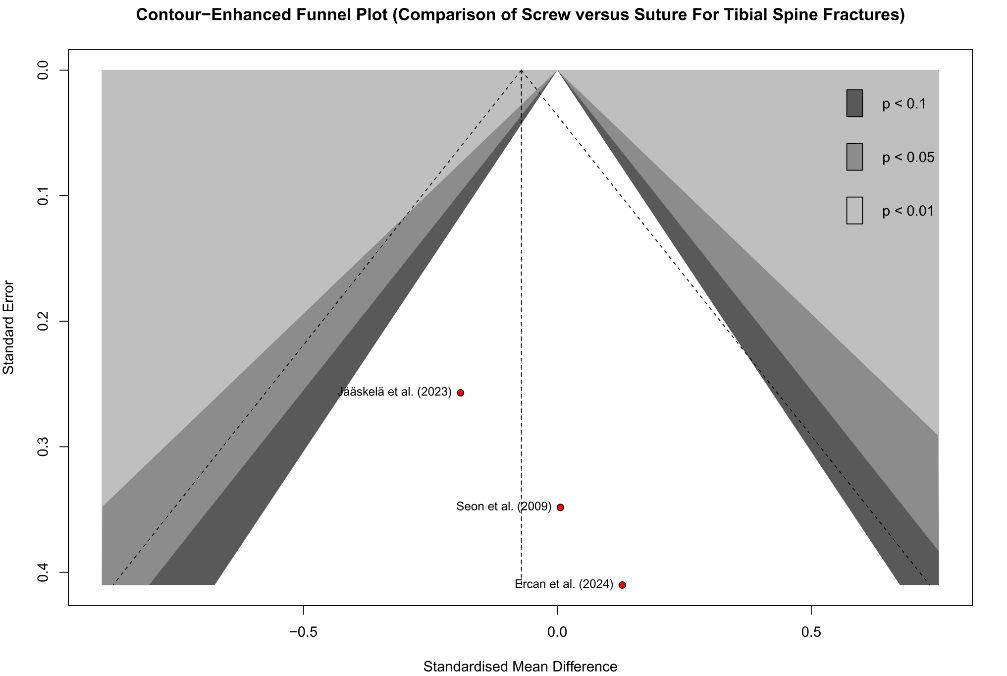


**Figure S4.** Bubble plot (scatter plot) of meta-regression between the mean age of participants and the functional outcome measured by the IKDC score.


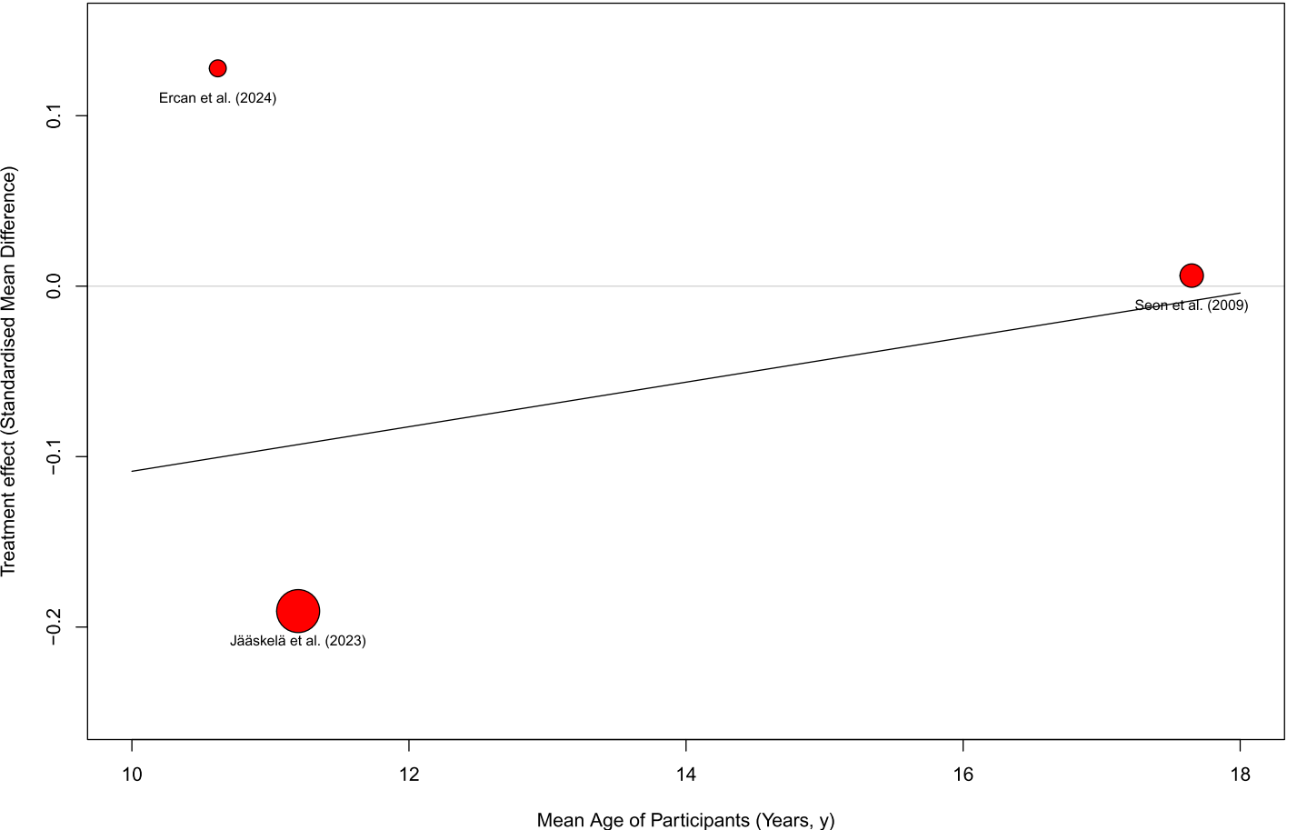


**Figure S5.** Bubble plot (scatter plot) of meta-regression between mean fracture severity (Meyers–McKeever classification) and the functional outcome measured by the IKDC score.


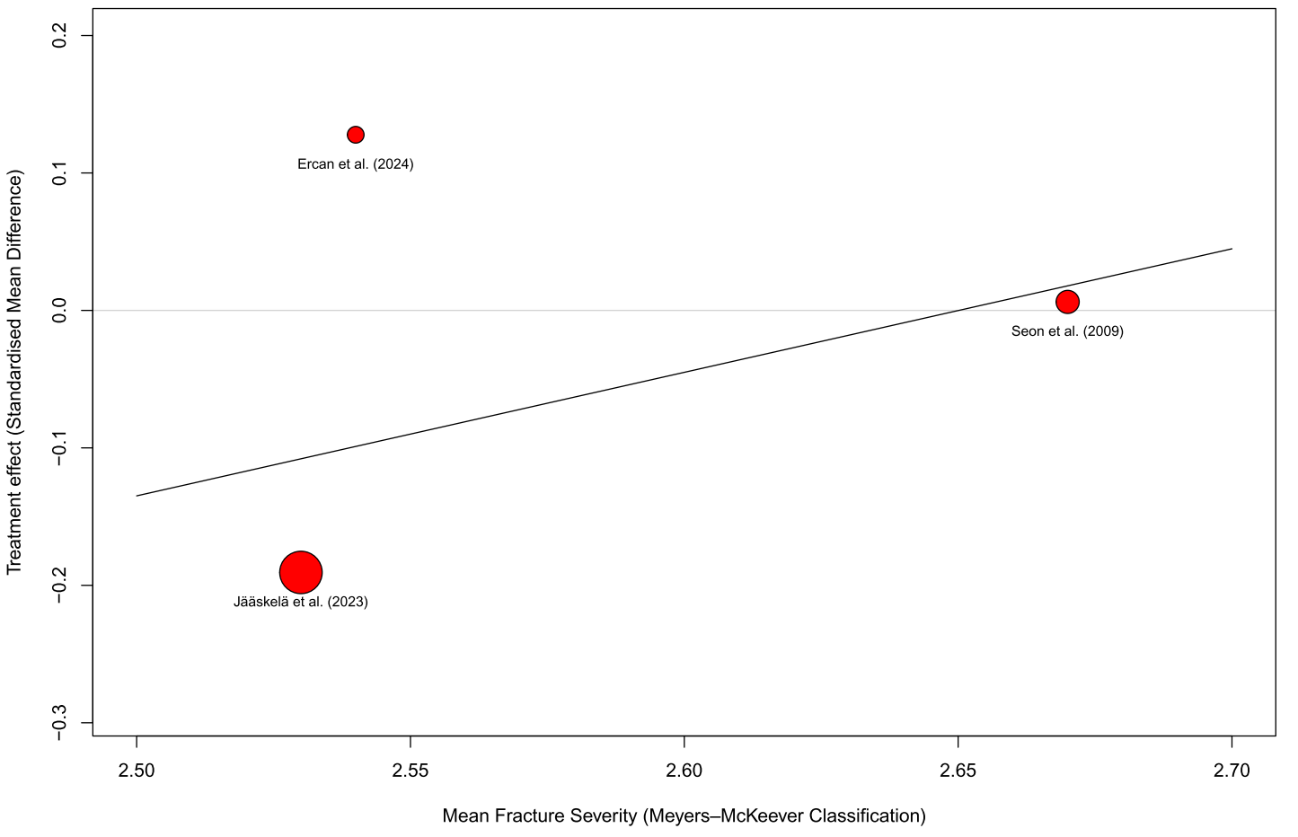


**Figure S6.** Bubble plot (scatter plot) of meta-regression between the mean proportion of patients treated arthroscopically and the functional outcome measured by the IKDC score.


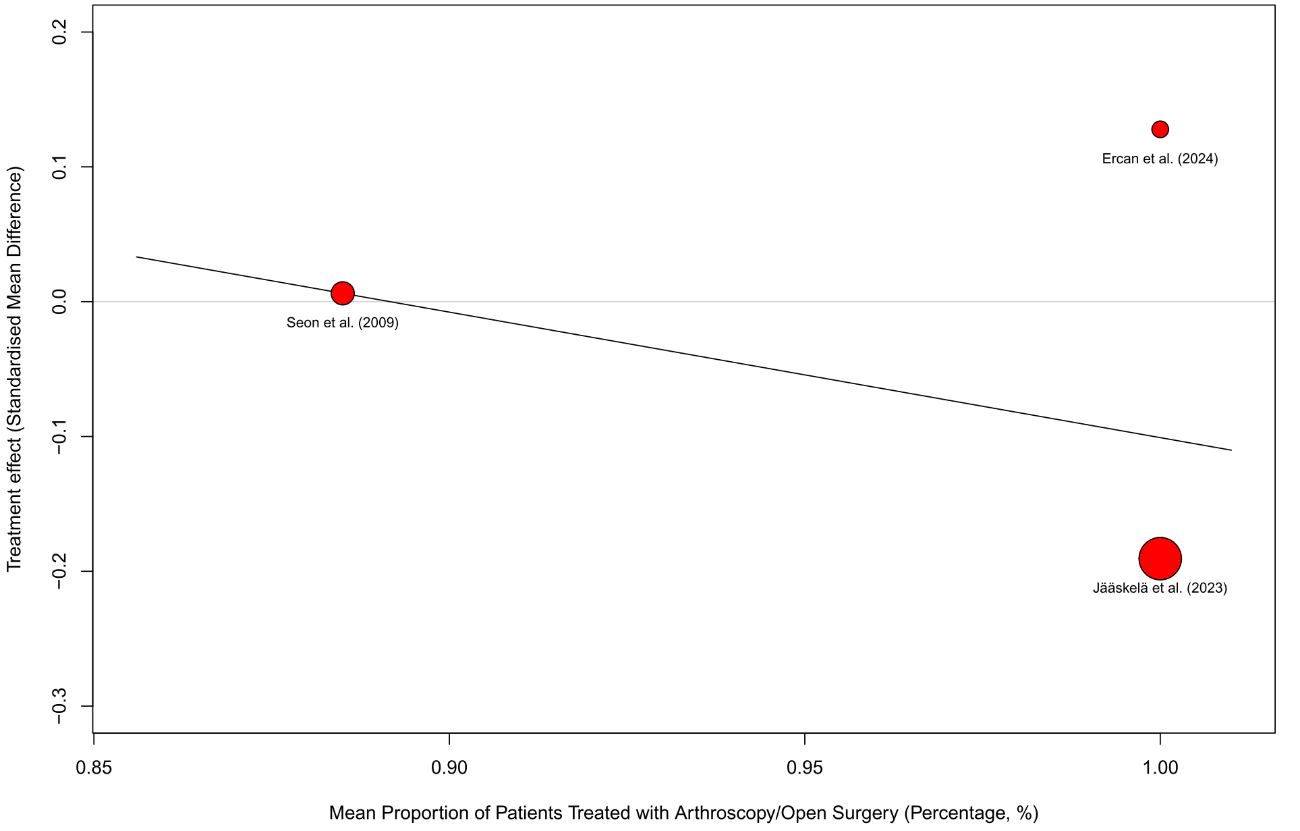


**Figure S7.** Bubble plot (scatter plot) of meta-regression between the mean proportion of male patients and the functional outcome measured by the IKDC score.


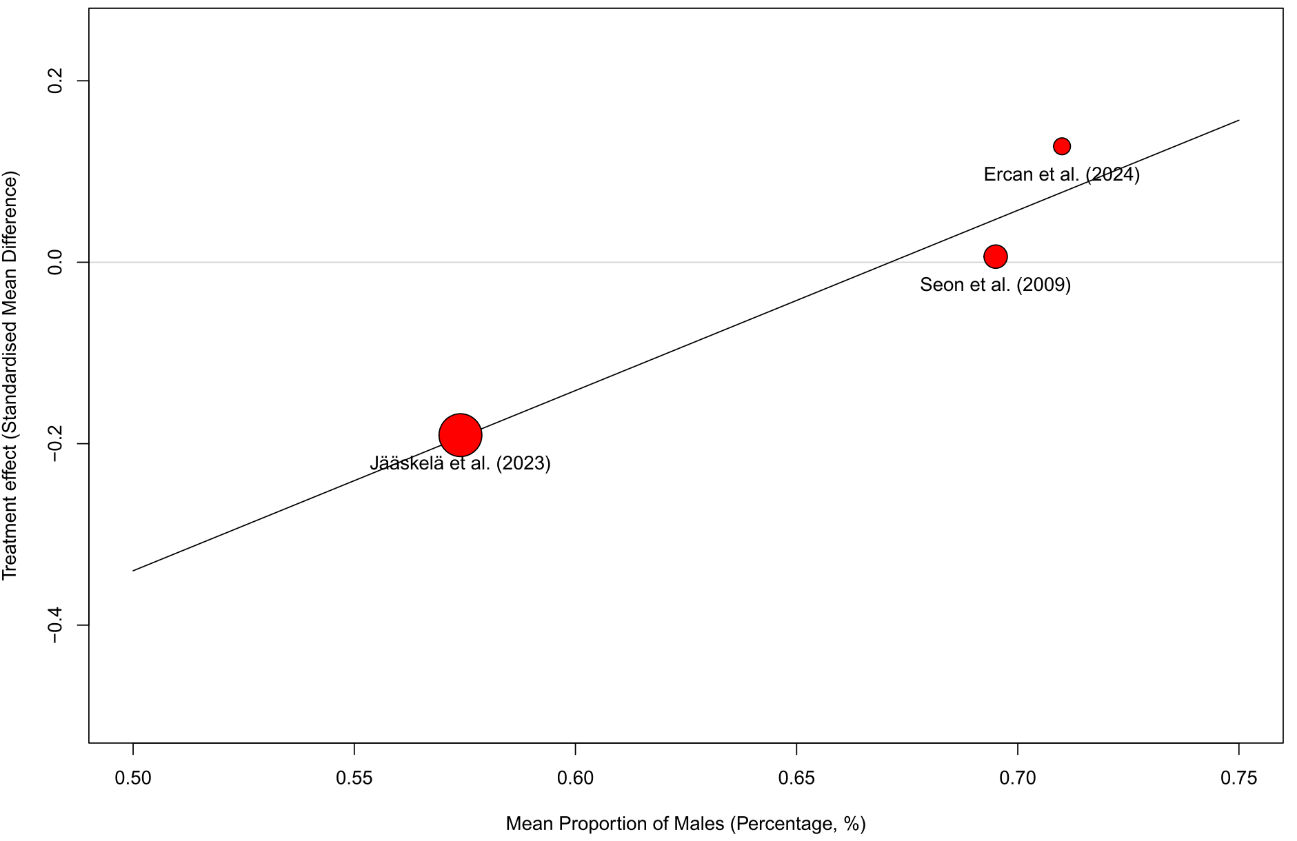


**Figure S8.** Forest plot of the meta-analysis comparing operative time (in minutes).


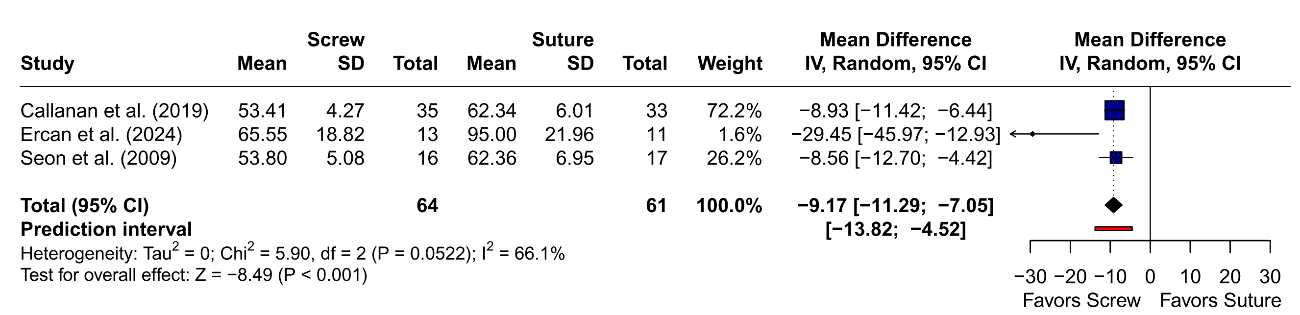


**Figure S9.** Leave-one-out sensitivity analysis for operative time.


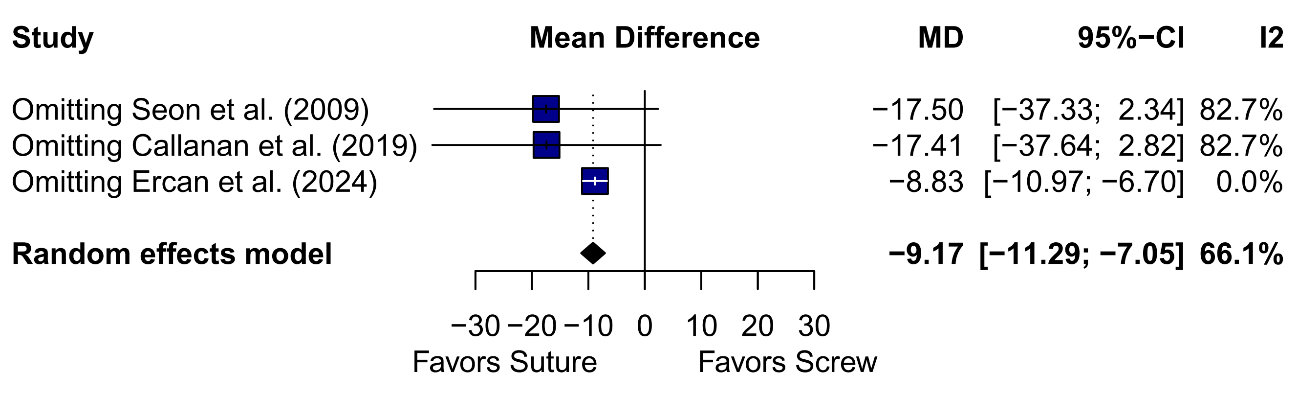


**Figure S10.** Forest plot of the relative risk of knee instability (Lachman test).


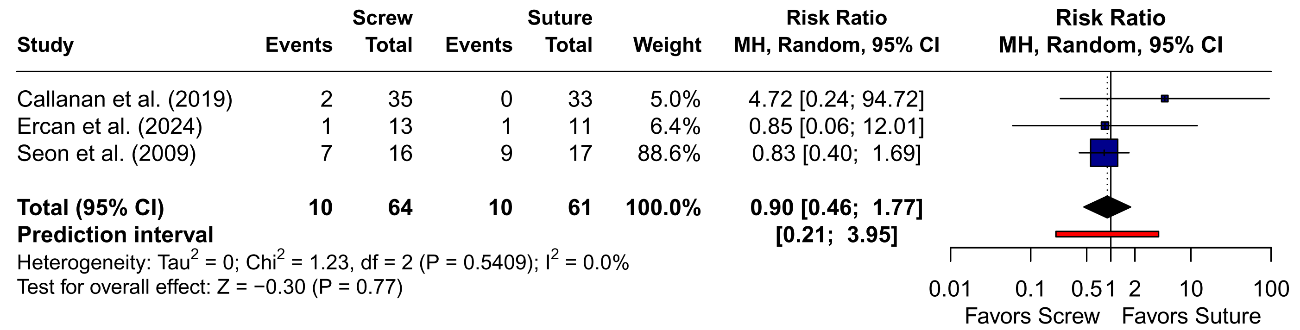


**Figure S11.** Leave-one-out sensitivity analysis for knee instability (Lachman test).


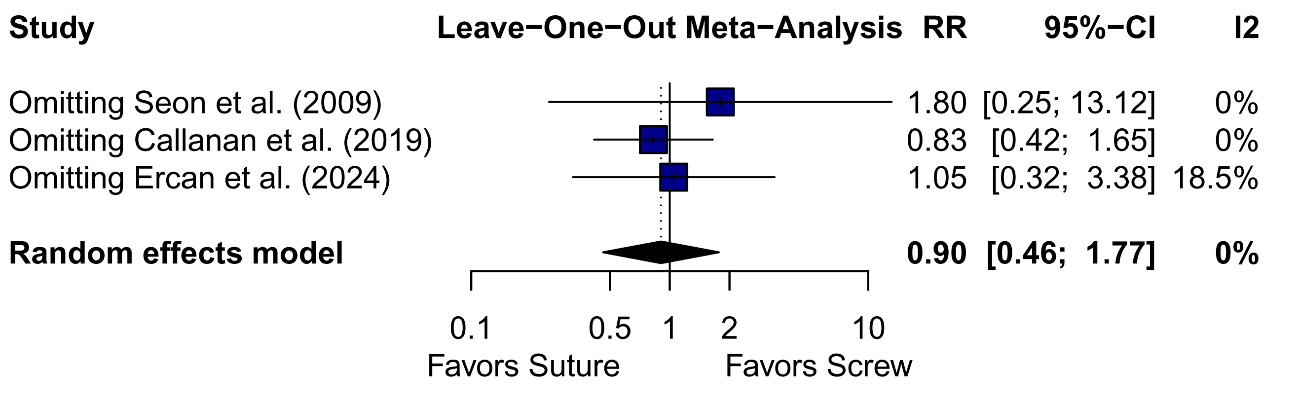


**Figure S12** Forest plot comparing the risk of failure to return to sport.


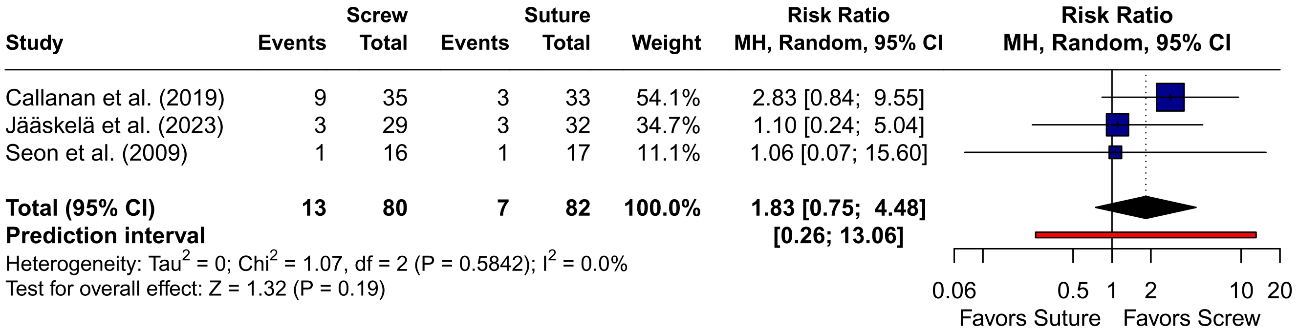


**Figure S13.** Leave-one-out sensitivity analysis for failure to return to sport.


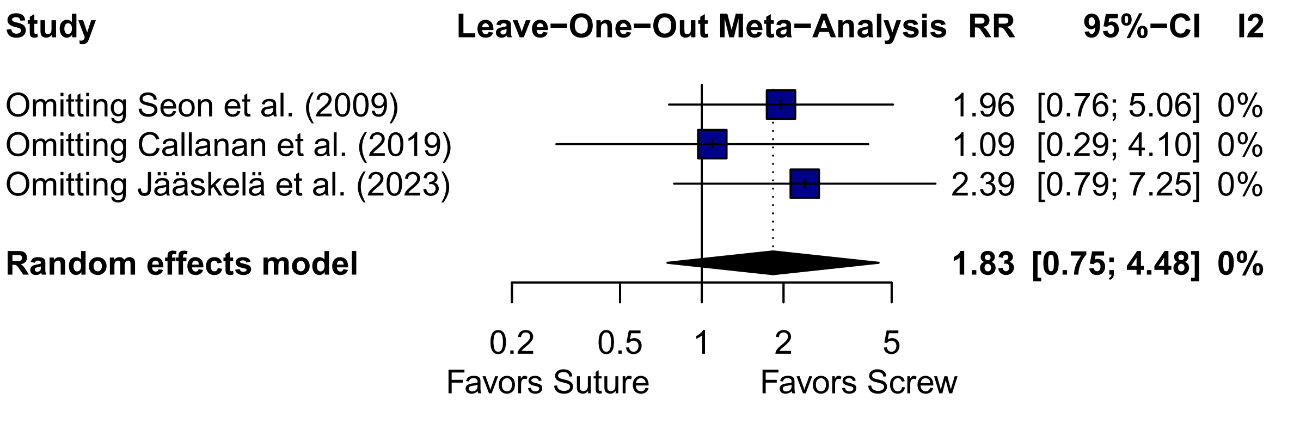


**Figure S14.** Forest plot of the relative risk of reoperations.


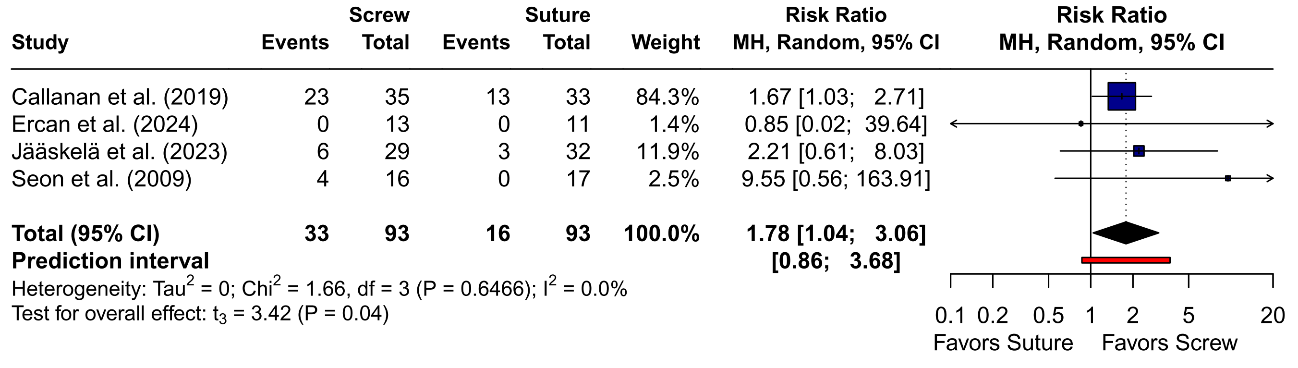


**Figure S15.** Leave-one-out sensitivity analysis for reoperations.


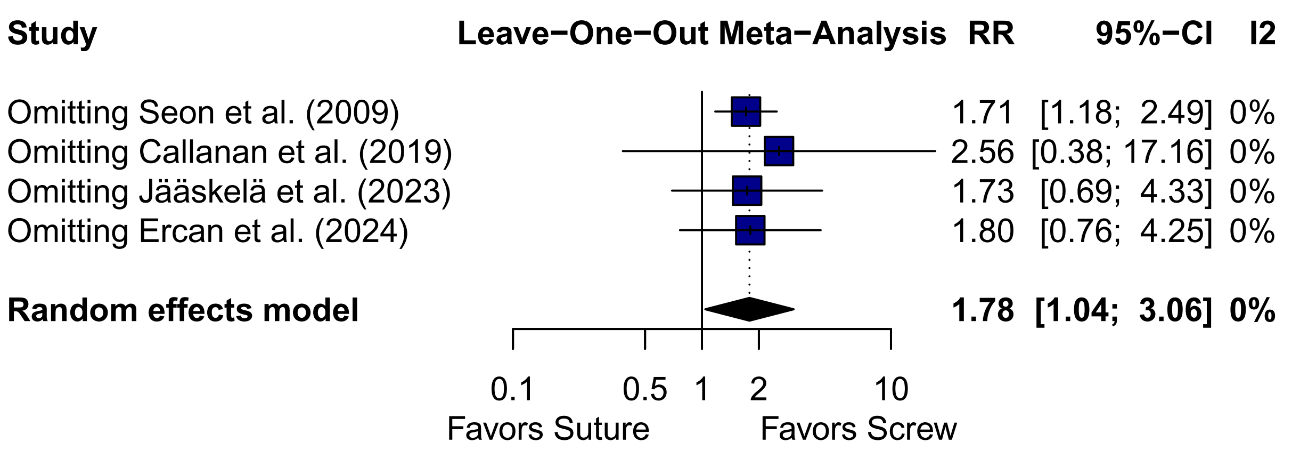


**Figure S16.** Forest plot comparing the relative risk of implant removal.


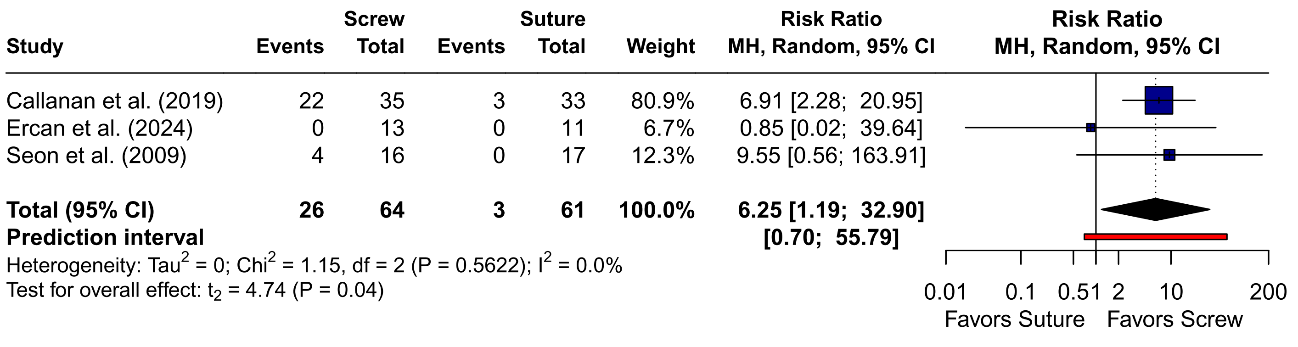


**Figure S17.** Leave-one-out sensitivity analysis for implant removal.


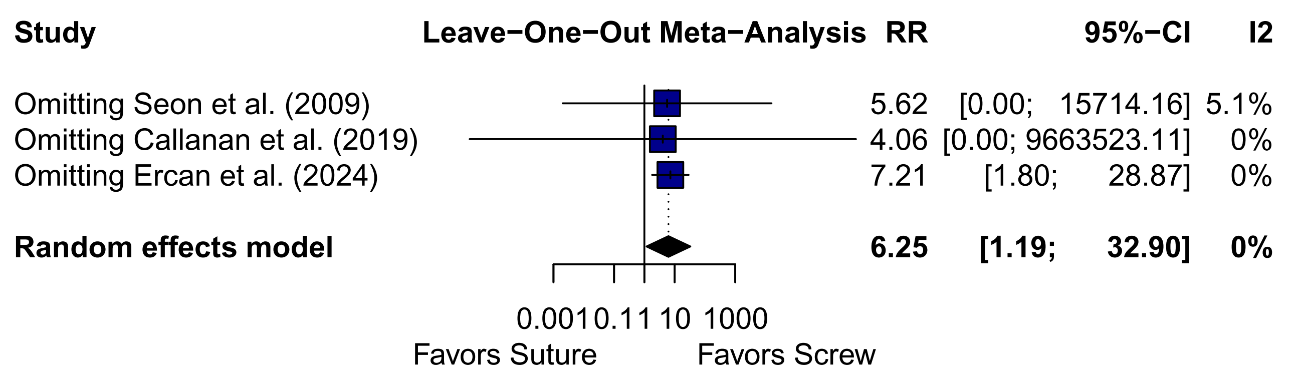


| **Table S1.** Meta-regression between the mean age of participants and the functional outcome measured by the IKDC score. | | | | |
| --- | --- | --- | --- | --- |
|  | Effect estimates | p-value | I² (%) | Test of residual heterogeneity |
| Mean age (Function) | | | | |
| Intercept | -0.23 | 0.77 | 0% | p = 0.83 |
| Mean age | **0.013** | **0.83** |  |  |

| **Table S2.** Meta-regression between mean fracture severity (Meyers–McKeever classification) and the functional outcome measured by the IKDC score. | | | | |
| --- | --- | --- | --- | --- |
|  | Effect estimates | p-value | I² (%) | Test of residual heterogeneity |
| Mean age (Function) | | | | |
| **Intercept** | -0.23 | 0.75 | 0% | P = 0.52 |
| **Mean Fracture**  **Severity** | **0.89** | **0.30** |  |  |

| **Table S3.** Meta-regression between the mean proportion of patients treated arthroscopically and the functional outcome measured by the IKDC score. | | | | |
| --- | --- | --- | --- | --- |
|  | Effect estimates | p-value | I² (%) | Test of residual heterogeneity |
| Mean age (Function) | | | | |
| Intercept | 0.83 | 0.81 | 0% | p = 0.51 |
| Mean age | **-0.93** | **0.79** |  |  |

| **Table S4.** Meta-regression between the mean proportion of male patients and the functional outcome measured by the IKDC score. | | | | |
| --- | --- | --- | --- | --- |
|  | Effect estimates | p-value | I² (%) | Test of residual heterogeneity |
| Mean age (Function) | | | | |
| Intercept | -1.33 | 0.47 | 0% | p = 0.86 |
| Proportion of male | **1.98** | **0.49** |  |  |
